# Clinical Significance of Circulating Tumor Cells in the Portal Vein of Patients with Hepatocellular Carcinoma Undergoing Anatomical Liver Resection

**DOI:** 10.1245/s10434-025-18295-5

**Published:** 2025-09-09

**Authors:** Daisuke Takei, Tsuyoshi Kobayashi, Michinori Hamaoka, Keiso Matsubara, Ko Oshita, Yosuke Namba, Takeshi Tadokoro, Yuka Tanaka, Shintaro Kuroda, Hideki Ohdan

**Affiliations:** 1 Department of Gastroenterological and Transplant Surgery, Graduate School of Biomedical and Health Sciences, Hiroshima University, Hiroshima, Japan; 2 Department of Gastroenterological Surgery, National Hospital Organization, Shikoku Cancer Center, Matsuyama, Japan

**Keywords:** Hepatocellular carcinoma, Circulating tumor cells, Glypican-3, Portal vein invasion, PD-L1

## Abstract

**Background:**

Hepatocellular carcinoma (HCC) frequently invades the portal vein, leading to early recurrence and a poor prognosis. However, the mechanisms underlying this invasion remain unclear. In this study, we aimed to detect portal vein circulating tumor cells (CTCs) using a Glypican-3-positive detection method and evaluate their prognostic significance.

**Methods:**

This prospective study included 146 patients with HCC who underwent open anatomical hepatectomy. Blood samples from the peripheral, portal, and hepatic veins were collected intraoperatively, and CTCs were detected using magnetic enrichment and flow cytometry. Programmed cell death ligand 1 (PD-L1) expression in portal CTCs was evaluated in 40 patients.

**Results:**

Portal vein CTCs were detected in 45.8% of cases, more frequently than hepatic vein CTCs (*p* = 0.031). Portal vein CTC positivity correlated with microscopic portal vein invasion (*p* < 0.001), poorer disease-free survival (*p* = 0.014), and overall survival (*p* = 0.001). PD-L1-positive portal vein CTCs were observed in 13 of 40 cases (32.5%) and were significantly associated with poorer prognosis (*p* = 0.001).

**Conclusions:**

Portal vein CTCs are significant predictors of portal vein invasion, recurrence, and survival in HCC. PD-L1-positive portal vein CTCs may contribute to immune evasion and tumor progression. These findings suggest that portal vein CTCs are valuable prognostic biomarkers and potential therapeutic targets.

**Supplementary Information:**

The online version contains supplementary material available at 10.1245/s10434-025-18295-5.

Hepatocellular carcinoma (HCC) is known to frequently invade the portal vein, and portal vein invasion (PVI) is considered one of the most powerful prognostic factors.^1,2^ Although HCC is speculated to metastasize intrahepatically and systemically via the portal vein, the mechanism by which HCC cells easily invade the portal vein or hepatic vein remains unclear, and few reports have described the presence of cancer cells in the portal blood. Tumor invasion into the portal vein, and even microscopic PVI, is associated with an increased risk of early recurrence. However, it is difficult to microscopically detect PVI before HCC treatment.^3,4^ In addition, few reports have described the mechanism of cancer cell invasion into the portal vein and circulating tumor cells (CTCs) in the portal blood.^5^

Microvascular invasion of the portal vein is a potential source of intrahepatic metastasis.^6^ However, the detailed mechanism by which HCC cells invade the portal vein and form thrombi has not yet been elucidated. Tanaka et al. suggested that tumor pressure increases with capsule formation and that the pressure gradient between the tumor and the portal vein may contribute to the dissemination of tumor cells into the portal vein.^7^ Toyosaka et al. investigated the pericapsular vascular network of resected HCC specimens and proposed that vascular channels within the tumor capsule were continuous with the portal vein in encapsulated HCCs.^8^ They demonstrated that radiopaque media infused into the tumors flowed immediately into the surrounding portal vessels with minimal resistance.^8^ Although these findings support the role of the portal vein as an efferent vessel, the mechanism underlying the high frequency of portal invasion in HCC remains unclear. Similarly, the mechanisms by which CTCs—a key factor in hematogenous metastasis—survive in the bloodstream and initiate metastasis are not fully understood. There is little theoretical foundation explaining the dynamics of HCC progression into tumor thrombus within the portal vein.

Direct evidence linking portal vein CTCs with vascular invasion or metastasis, particularly postsurgical recurrence, remains limited. This study aimed to detect CTCs in the portal vein using our previously developed method for identifying Glypican-3-positive CTCs and to investigate their clinicopathological significance and clinical outcomes.^9^ In a study by Hamaoka et al.,^9^ peripheral blood CTCs positive for Glypican-3 were significantly associated with microscopic PVI and poorer overall survival (OS).

In the present study, in addition to examining portal vein CTCs, we also evaluated programmed cell death ligand 1 (PD-L1). The prognostic and predictive value of PD-L1^+^ CTCs has been reported in various cancers.^10^ In HCC, PD-L1-expressing CTCs have been proposed as potential prognostic and predictive markers for immune checkpoint inhibitors.^11–13^ However, most patients in these studies had unresectable advanced disease, and no reports have assessed whether PD-L1⁺ CTCs are prognostic in patients undergoing hepatic resection. Furthermore, no studies have evaluated PD-L1 expression in CTCs derived from portal blood. In this study, we assessed PD-L1 expression in CTCs collected from different vascular compartments in patients undergoing resection for HCC.

## Methods

### Study Design

This prospective single-institution study was conducted in accordance with the 1975 Declaration of Helsinki. The study was approved by the institutional review board of Hiroshima University, Hiroshima, Japan (approval number: E2016-0320) and registered with the University Hospital Medical Information Network Clinical Trial Registry (UMIN-CTR), Japan (registration number: UMIN000025989).

Among 383 Japanese patients with HCC who underwent initial hepatectomy between April 2015 and December 2022 at our institution, 198 underwent open anatomical hepatectomy. The inclusion criteria of this study were as follows: (1) pathological diagnosis of HCC, (2) curative treatment with open anatomical hepatectomy, and (3) availability of CTC assessment data. The exclusion criteria were as follows: (1) missing data, (2) macroscopic residual disease after surgery (R2 resection), and (3) absence of CTC assessment. Ultimately, 146 patients with complete CTC data were enrolled (Supplementary Fig. 1). Written informed consent was obtained from all the patients.

Among these, 40 patients with an assessment of PD-L1 expression on the CTC surface conducted after July 2020 were included in the subgroup PD-L1-CTC analysis.

### Liver Resection

The indications for liver resection have been previously described.^14^ According to Clinical Practice Guidelines for Hepatocellular Carcinoma established by The Japan Society of Hepatology, hepatic resection is considered one of the standard treatments for PVI limited to the first-order branch; therefore, even cases with PVI are considered surgical candidates.^15^ The type of liver resection was decided based on the functional reserve of the liver and the location of the tumor. The liver functional reserve was assessed based on the Child-Pugh classification and the indocyanine green retention rate at 15 min (ICG-R15). Child-Pugh class C was considered a contraindication for partial hepatectomy.

Portal vein invasion was classified as either microscopic or macroscopic. Microscopic PVI refers to tumor infiltration into small peripheral branches, detected only by histopathological examination, whereas macroscopic PVI represents gross involvement of the portal vein branches, visible on imaging or during surgery. Furthermore, because tumor thrombus is considered a subtype of macroscopic PVI, it was included in the macroscopic PVI category in this study. Carr et al.^16^ reported that cases with macroscopic PVI frequently exhibit coexisting microscopic PVI. Therefore, when macroscopic PVI is confirmed visually, microscopic PVI is also presumed to be present, provided it is pathologically verified.

The types of liver resection were selected as follows: anatomical resection (segmentectomy, sectionectomy, or hemihepatectomy) was performed when liver function allowed, whereas limited resection was performed in patients with insufficient hepatic functional reserve. The types of liver resection performed are shown in Supplementary Table 1.

The follow-up evaluation after surgery consisted of clinical physical examinations, blood chemistry tests, and measurements of tumor marker levels, including alpha-fetoprotein (AFP) and des-gamma-carboxy prothrombin (DCP), every month for 2 years. After 2 years, patients were assessed every 3 months. Patients were examined using ultrasonography every 3 months and computed tomography every 6 months. When recurrence was indicated by any of these examinations, patients underwent hepatic angiography. Repeat hepatectomy was indicated if all recurrent tumors could be resected within the hepatic functional reserve. If they could not be resected within the hepatic functional reserve, they underwent percutaneous radiofrequency ablation or transarterial chemoembolization. The median follow-up period for survivors was 40.4 (range 12–107) months.

### Sample Collection, Identification, and Analysis of CTCs

For all patients, an 8.0 mL blood sample was collected as follows. Peripheral blood was collected immediately before the surgery. Portal and hepatic vein samples were obtained by intraoperative ultrasound-guided direct venous puncture before resection of the primary HCC tumor and after mobilization of the liver during the operation.

CTCs originating from the peripheral (peCTC), portal (poCTC), and hepatic venous blood (hvCTC) were detected by using previously described methods.^6^ Briefly, CTCs were isolated using a combination of magnetic cell sorting (MACS) and flow cytometry (FCM).

Blood samples (8.0 mL) were collected into BD Vacutainer CPT Cell Preparation Tubes containing sodium citrate, a polyester gel, and a density gradient medium. Samples were centrifuged at 1800×*g* for 20 min at 25 °C, and the mononuclear cell layer (including CTCs) was harvested. All samples were processed within 120 min of blood collection.

The mononuclear fraction was resuspended and washed with MACS buffer, then incubated with APC-conjugated mouse antihuman GPC3 monoclonal antibodies (clone #307801) at 4 °C for 30 min in the dark. Subsequently, anti-APC MicroBeads (Miltenyi Biotec) were added and incubated for 15 min. GPC3-positive cells were then magnetically enriched using the autoMACS Separator.

The enriched cells were further analyzed by FCM. Cells were stained with antibodies against CD45 and CD235a and treated with 7-AAD to exclude dead cells. Cells characterized as GPC3⁺CD45^−^CD235a^−^7-AAD^−^ were identified as CTCs. Flow cytometric analysis was performed using the BD FACS Canto II system, and data were analyzed with FlowJo software (version 7.6.5).

The number of CTCs in the enriched samples was enumerated via FCM by independent technicians who were blinded to all clinicopathological factors and patient outcomes.

### Analysis of PD-L1 Expression on CTCs

The FCM was used to evaluate whether PD-L1 was expressed in CTC. When identifying CTC using a previously described method, mouse antihuman PD-L1 antibodies (Becton, Dickinson and Company, NJ) were simultaneously stained. The remaining GPC3-negative cell population, using immunomagnetic positive selection, was used as the control. This cell population was analyzed by FCM as a control for PD-L1-negative cells. The highest point of fluorescence intensity of the cell population was considered the cutoff line, and cells showing fluorescence intensity higher than the cutoff line were considered PD-L1-positive cells.

### Statistical Analysis

All statistical analyses were performed using JMP®Pro 18 software (SAS Institute Inc., Cary, NC). The Wilcoxon rank-sum test was used to compare continuous variables. Categorical variables were compared using the chi-square test or Fisher’s exact probability test, as appropriate. Disease-free survival (DFS) was defined as the time from surgery to the first documented recurrence or death, whichever occurred first. Overall survival was defined as the time from surgery to death from any cause. Disease-free survival rates were calculated by using the Kaplan–Meier method and were compared by using the log-rank test. A receiver operating characteristic (ROC) curve was used to determine the cutoff value for each continuous variable. In multivariate analysis, a multiple logistic regression model was used to identify independent predictors of microscopic PVI, and a Cox proportional hazards model was used to identify independent predictors of DFS and OS. Only variables that were statistically significant in the univariate analysis were included in the multivariate analysis. Statistical significance was set at *p* values < 0.05.

## Results

### Distribution of Circulating GPC3-Positive Cells in the Peripheral, Portal, and Hepatic Veins

The patient demographics and clinicopathological characteristics are summarized in Supplementary Table 1. Preoperative treatment consisted of transarterial chemoembolization in 11 cases and radiofrequency ablation in one case; no patients received chemotherapy or immune checkpoint inhibitor therapy. The number of CTCs was not affected by preoperative treatment; in other words, there was no difference in CTC count between patients who received preoperative treatment and those who did not.

From 8-mL blood samples, a median (interquartile range) of three (1–6), four (1–8), and three (1–6) GPC3-positive CTCs were detected in the peCTC, poCTC, and hvCTC, respectively (Supplementary Fig. 2). The number of CTCs was significantly higher in the portal vein than in the hepatic vein (*p* = 0.011). The optimal cutoff value for each CTC type (peCTC, poCTC, and hvCTC) was determined to be five, based on ROC analysis of CTC count for predicting microscopic PVI (Supplementary Fig. 3). CTC positivity in the peripheral, portal, and hepatic venous blood was observed in 54 of 146 cases (36.9%), 67 of 146 cases (45.8%), and 49 of 146 cases (33.5%), respectively. Positivity for CTC was significantly higher in portal blood than in hepatic venous blood (*p* = 0.031). The CTC positivity rate was not significantly different between peripheral and portal blood samples (*p* = 0.122).

### Associations Between Patient Characteristics and the Presence of peCTC, poCTC, and hvCTC

A comparison of the clinicopathological factors in patients with peCTC levels above and below five is described in Supplementary Table 2. Patients positive for peCTC had significantly more tumors and larger tumor diameters than did those negative for peCTC (*p* = 0.017). Furthermore, microscopic invasion of the portal vein and hepatic vein was significantly more frequent in patients with positive peCTC than in those with negative peCTC (*p* < 0.001 and *p* = 0.011, respectively). Macroscopic PVI was also significantly more frequent in the peCTC-positive group (*p* = 0.016).

A comparison of the clinicopathological factors in patients with poCTCs above and < 5 poCTCs is described in Table 1. Although there were no significant differences in either tumor diameter or the number of tumors between patients with positive poCTC and those with negative poCTC (*p* = 0.146 and *p* = 0.608, respectively), patients with positive poCTC had significantly more microscopic PVI than those with negative poCTC (*p* < 0.001). In contrast, microscopic hepatic vein invasion (HVI) did not significantly differ between the two groups. Among patients with positive poCTC, macroscopic PVI showed a trend toward significance (*p* = 0.06).

**Table 1:** Comparison of clinicopathologic factors in patients with poCTC above and below 5

Variables	poCTC ≥ 5 (*n* = 67)	poCTC < 5 (*n* = 79)	*p*
Age (years)^*^	72 (48–87)	74 (48–89)	0.205
Male: *n* (%)	55 (82%)	60 (76%)	0.366
BMI (kg/m^2^)^*^	23 (16.7–30.3)	23.4 (19.1–33.1)	0.316
HBV: *n* (%)	6 (9%)	10 (13%)	0.475
HCV: *n* (%)	27 (40%)	33 (42%)	0.856
ICGR15 (%)^*^	12.0 (2.6–37.7)	12.3 (2.5–41.1)	0.754
Child–Pugh grade B: *n* (%)	4 (6%)	4 (5%)	0.814
AFP (ng/mL)^*^	7.2 (1.4–290700)	5.8 (0.7–99430)	0.233
DCP (mAU/mL)^*^	435 (3.4–124040)	103 (10–82160)	0.141
Tumor number^*^	1 (1–20)	1 (1–6)	0.608
Tumor size (mm)^*^	35 (12–200)	30 (10–130)	0.146
Macrocsopic Portal vein invasion: *n* (%)	5 (7%)	1 (1%)	0.06
Macroscopic Hepatic vein invasion: *n* (%)	3 (4%)	4 (5%)	0.868
Microcsopic Portal vein invasion: *n* (%)	25 (37%)	8 (10%)	< 0.001
Microscopic Hepatic vein invasion: *n* (%)	9 (13%)	6 (8%)	0.247
Major hepatectomy	11 (16%)	11 (14%)	0.674
Surgical margin (mm)	2 (0–30)	3 (0–40)	0.893
Estimated blood loss (g)	536 (35–3385)	473 (20–3242)	0.264

*poCTC* portal vein circulating tumor cell, *BMI* body mass index, *HBV* hepatitis B virus, *HCV* hepatitis C virus, *ICGR15* indocyanine green retention rate at 15 min, *AFP* α-fetoprotein, *DCP* des-γ-carboxy prothrombin

*Median (range)

A comparison of clinicopathological factors in patients with hvCTC levels above and below five is shown in Supplementary Table 3. There were no significant differences in tumor diameter, number of tumors, or microscopic and macroscopic portal or HVI between patients positive and negative for hvCTCs.

### Relationship Between Macroscopic and Microscopic PVI and Their Association with CTC

The number of CTCs at each vascular site, stratified by the presence or absence of macroscopic PVI, is presented in Supplementary Table 4. The number of peCTCs was significantly higher in patients with macroscopic PVI, whereas no significant differences were observed for poCTCs and hvCTCs. However, all CTC types tended to be more numerous in the presence of macroscopic PVI.

The relationship between macroscopic and microscopic PVI is shown in Supplementary Table 5. The sensitivity of macroscopic PVI for detecting microscopic PVI was 0.152, whereas the specificity was 0.991.

Supplementary Table 6 illustrates the association between the positivity rates of CTCs at each site and the presence of microscopic or macroscopic PVI. In cases positive for microscopic PVI, peCTCs and poCTCs showed significantly higher positivity rates (*p* < 0.001 for both). Additionally, the positivity rate of peCTCs was significantly higher in cases with macroscopic PVI (*p* = 0.026).

### Correlation Between poCTCs and the Incidence of Microscopic PVI

**Fig. 1:**
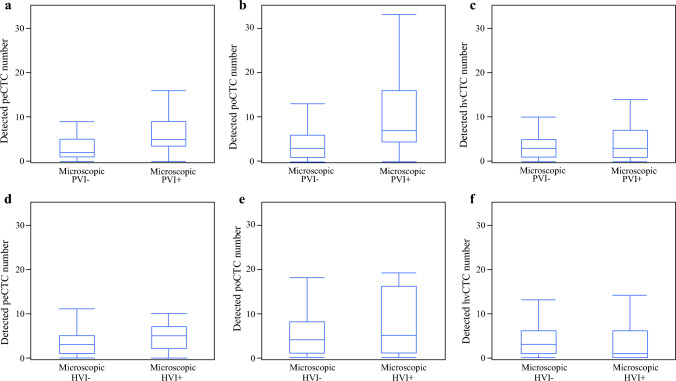
Comparison of the number of CTCs with or without microvascular invasion at different sites. **a** Detected number of peCTC in microscopic PVI-positive patients was significantly higher than in microscopic PVI-negative patients (5 [0–18] vs. 2 [0–77], *p* < 0.001). **b** Detected number of poCTC in microscopic PVI-positive patients was significantly higher than in microscopic PVI-negative patients (7 [0–43] vs. 3 [0–29], *p* < 0.001). **c** Detected number of hvCTC in microscopic PVI-positive patients was similar to that of microscopic PVI-negative patients (3 [0–14] vs. 3 [0–51], *p* = 0.261). **d** Detected number of peCTC in microscopic HVI-positive patients was similar to that of microscopic HVI-negative patients (5 [0–18] vs. 3 [0–77], *p* = 0.1). **e** Detected number of poCTC in microscopic HVI-positive patients was similar to that of microscopic HVI-negative patients (5 [0–43] vs. 4 [0–33], *p* = 0.329). **f** Detected number of hvCTC in microscopic HVI-positive patients was similar to that of microscopic HVI-negative patients (1 [0–14] vs. 3 [0–51], *p* = 0.36)

We examined the incidence of microscopic PVI and HVI. As shown in Fig. 1a and b, peCTCs and poCTCs were detected significantly more frequently in patients with microscopic PVI than in those without microscopic PVI. Conversely, hvCTCs were equally detected regardless of microscopic PVI (Fig. 1c). peCTCs, poCTCs, and hvCTCs were detected regardless of the microscopic HVI (Fig. 1d–f).

The number of CTCs according to the status of microscopic PVI and HVI, categorized into four groups—Both, only PVI, only HVI, and None—is presented in Supplementary Table 7. In peripheral blood, the number of CTCs was significantly higher in the Both (*p* = 0.005) and only PVI (*p* = 0.001) groups than in the None group, with no significant differences observed among the other groups. In portal vein blood, CTC counts were also significantly higher in the Both (*p* = 0.025) and only PVI (*p* = 0.001) groups than in the None group; no other significant differences were noted. In hepatic vein blood, no significant differences in CTC counts were observed among the groups.

Next, we examined the factors involved in microscopic PVI. Univariate analysis showed that AFP level ≥ 60 ng/mL (*p* < 0.001), DCP value ≥ 100 mAU/mL (*p* = 0.016), tumor size ≥ 30 mm (*p* = 0.003), peCTC ≥ 5 (*p* < 0.001), and poCTC ≥ 5 (*p* < 0.001) were associated with microscopic PVI. Subsequent multivariate analysis identified AFP level ≥ 60 ng/mL (*p* < 0.001), peCTC ≥ 5 (*p* < 0.001), and poCTC ≥ 5 (*p* = 0.012) as independent risk factors for microscopic PVI (Table 2).

**Table 2:** Factors involved in microscopic portal vein invasion

Variables	*n*	Univariate analysis		Multivariate analysis	
		OR (95% CI)	*p*	OR (95% CI)	*p*
Age, ≥ 75 years	63	1.111 (0.504–2.425)	0.791		
Gender, male	115	1.001 (0.402–2.748)	0.997		
BMI, ≥ 24.5 kg/m^2^	53	1.379 (0.616–3.038)	0.428		
HBV	16	0.456 (0.069–1.753)	0.277		
HCV	60	1.729 (0.790–3.807)	0.169		
ICGR15, ≥ 10%	92	1.035 (0.467–2.368)	0.932		
Child–Pugh grade, B	8	2.160 (0.423–9.320)	0.327		
AFP, ≥ 60 ng/mL	33	9.596 (4.018–23.991)	< 0.001	16.7 (5.148–68.199)	< 0.001
DCP, ≥ 100 mAU/mL	80	2.714 (1.193–6.644)	0.016	1.631 (0.514–5.442)	0.406
Multiple tumors	50	1.335 (0.589–2.957)	0.482		
Tumor size, ≥ 30 mm	89	3.701 (1.502–10.537)	0.003	1.403 (0.416–5.083)	0.586
No. CTC in peripheral blood, ≥ 5 cells	54	6.083 (2.666–14.774)	< 0.001	7.25 (2.329–27.833)	< 0.001
No. CTC in portal vein blood, ≥ 5 cells	67	5.282 (2.267–13.505)	< 0.001	4.015 (1.343–13.595)	0.012
No. CTC in hepatic vein blood, ≥ 5 cells	49	1.642 (0.731–3.637)	0.226		

*BMI* body mass index, *HBV* hepatitis B virus, *HCV* hepatitis C virus, *ICGR15* indocyanine green retention rate at 15 min, *AFP*α-fetoprotein, *DCP* des-γ-carboxy prothrombin, *CTC* circulating tumor cell

### Associations Between poCTC and HCC Prognostic Factors

**Fig. 2:**
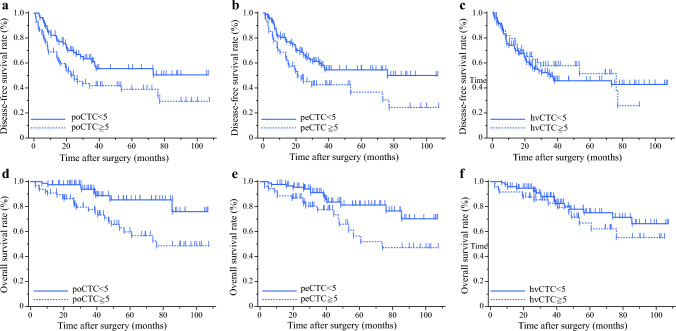
Survival after surgery by CTC counts by site of collection. **a** Comparison of overall survival rates after hepatectomy between the peCTC ≥ 5 group and the peCTC < 5 group. The overall survival rates were significantly lower in the peCTC ≥ 5 group than in the peCTC < 5 group (*p* = 0.014). **b** Comparison of disease-free survival rates after hepatectomy between the peCTC ≥ 5 group and the peCTC < 5 group. The disease-free survival rates were significantly lower in the peCTC ≥ 5 group than in the peCTC < 5 group (*p* = 0.001). **c** Comparison of overall survival rates after hepatectomy between the poCTC ≥ 5 group and the poCTC < 5 group. The overall survival rates were significantly lower in the poCTC ≥ 5 group than in the poCTC < 5 group (*p* = 0.013). **d** Comparison of disease-free survival rates after hepatectomy between the poCTC ≥ 5 group and the poCTC < 5 group. The disease-free survival rates were significantly lower in the poCTC ≥ 5 group than in the poCTC < 5 group (*p* = 0.012). **e** Comparison of overall survival rates after hepatectomy between the hvCTC ≥ 5 group and the hvCTC < 5 group. There was no significant difference in overall survival rates between the two groups (*p* = 0.657). **f** Comparison of disease-free survival rates after hepatectomy between the hvCTC ≥ 5 group and the hvCTC < 5 group. There was no significant difference in disease-free survival rates between the two groups (*p* = 0.27)

The follow-up period for patients in our study ranged from 2.9 to 107 months, with a median of 38 months. When evaluated with respect to poCTC, DFS in the poCTC-positive group was significantly lower than that in the poCTC-negative group (*p* = 0.014; Fig. 2a). In addition, the poCTC-positive group had a significantly lower OS rate than did the poCTC-negative group (*p* = 0.001; Fig. 2b). Similarly, for peCTC, DFS was significantly lower in the peCTC-positive group than in the peCTC-negative group (*p* = 0.013; Fig. 2c), and OS was significantly lower in the peCTC-positive group than in the peCTC-negative group (*p* = 0.012; Fig. 2d). With regard to hvCTC, there was no significant difference in DFS and OS between the hvCTC-positive and hvCTC-negative groups (*p* = 0.657 and *p* = 0.27, respectively; Fig. 2e and f).

Next, we sought to identify prognostic risk factors. In the univariate analyses, the significant prognostic factors for DFS included Child-Pugh grade B (*p* = 0.006), AFP ≥ 60 ng/mL (*p* = 0.015), DCP ≥ 100 mAU/mL (*p* = 0.019), multiple tumors (*p* = 0.001), tumor size ≥ 30 mm (*p* = 0.008), microscopic PVI (*p* < 0.001), microscopic HVI (*p* = 0.002), peCTC-positive (*p* = 0.014), and poCTC-positive (*p* = 0.016). Multivariate analysis identified multiple tumors (*p* < 0.001) and poCTC-positive (*p* = 0.043) as independent prognostic factors for DFS (Table 3).

**Table 3:** Prognostic factors for disease-free survival

Variables	*n*	Univariate analysis		Multivariate analysis	
		HR (95% CI)	*p*	HR (95% CI)	*p*
Age, ≥ 75 years	63	1.067 (0.667–1.707)	0.784		
Gender, Male	115	1.570 (0.826–2.984)	0.168		
BMI, ≥ 24.5 kg/m^2^	53	0.620 (0.370–1.038)	0.069		
HBV	16	0.546 (0.219–1.357)	0.193		
HCV	60	0.921 (0.573–1.480)	0.735		
ICGR15, ≥ 10%	92	1.439 (0.871–2.378)	0.155		
Child–Pugh grade, B	8	3.213 (1.382–7.465)	0.006	2.718 (1.093–6.756)	0.031
AFP, ≥ 60 ng/mL	33	1.873 (1.125–3.118)	0.015	1.582 (0.879–2.845)	0.125
DCP, ≥ 100 mAU/mL	80	1.778 (1.098–2.877)	0.019	1.365 (0.810–2.301)	0.241
Multiple tumors	50	2.196 (1.377–3.503)	0.001	2.420 (1.472–3.977)	< 0.001
Tumor size, ≥ 30 mm	89	1.983 (1.191–3.301)	0.008	1.607 (0.904–2.854)	0.105
Microscopic					
Portal vein invasion	33	2.415 (1.468–3.973)	< 0.001	1.183 (0.592–2.366)	0.632
Microscopic					
Hepatic vein invasion	15	2.757 (1.445–5.258)	0.002	1.846 (0.901–3.784)	0.093
No. CTC in peripheral blood, ≥ 5 cells	54	1.784 (1.120–2.840)	0.014	1.087 (0.604–1.956)	0.78
No. CTC in portal vein blood, ≥ 5 cells	67	1.774 (1.112–2.830)	0.016	1.782 (1.018–3.119)	0.043
No. CTC in hepatic vein blood, ≥ 5 cells	49	0.893 (0.544–1.468)	0.657		

*BMI* body mass index, *HBV* hepatitis B virus, *HCV* hepatitis C virus, *ICGR15* indocyanine green retention rate at 15 min, *AFP*α-fetoprotein, *DCP* des-γ-carboxy prothrombin, *CTC* circulating tumor cell

In univariate analyses, significant prognostic factors for OS included multiple tumors (*p* = 0.003), microscopic PVI (*p* = 0.026), microscopic HVI (*p* = 0.01), peCTC-positive (*p* = 0.015), and poCTC-positive (*p* = 0.003). Multivariate analysis identified multiple tumors (*p* = 0.006), microscopic HVI (*p* = 0.011), and poCTC-positive (*p* = 0.005) as independent prognostic factors for OS (Table 4).

**Table 4:** Prognostic factors for overall survival

Variables	*n*	Univariate analysis		Multivariate analysis	
		HR (95% CI)	*p*	HR (95% CI)	*p*
Age, ≥ 75 years	63	1.777 (0.865–3.649)	0.117		
Gender, Male	115	2.552 (0.777–8.384)	0.122		
BMI, ≥ 24.5 kg/m^2^	53	0.579 (0.260–1.289)	0.181		
HBV	16	0.820 (0.249–2.697)	0.744		
HCV	60	0.882 (0.431–1.805)	0.732		
ICGR15, ≥ 10%	92	2.036 (0.912–4.544)	0.082		
Child–Pugh grade, B	8	3.163 (0.955–10.476)	0.059		
AFP, ≥ 60 ng/mL	33	1.802 (0.852–3.808)	0.122		
DCP, ≥ 100 mAU/mL	80	1.457 (0.711–2.983)	0.303		
Multiple tumors	50	2.879 (1.429–5.803)	0.003	2.728 (1.319–5.639)	0.006
Tumor size, ≥ 30 mm	89	1.901 (0.878–4.114)	0.102		
Microscopic Portal vein invasion	33	2.290 (1.100–4.768)	0.026	0.975 (0.390–2.438)	0.958
Microscopic Hepatic vein invasion	15	3.210 (1.314–7.840)	0.01	3.727 (1.348–10.299)	0.011
No. CTC in peripheral blood, ≥ 5 cells	54	2.375 (1.180–4.778)	0.015	1.209 (0.515–2.839)	0.661
No. CTC in portal vein blood, ≥ 5 cells	67	3.323 (1.490–7.409)	0.003	3.473 (1.440–8.374)	0.005
No. CTC in hepatic vein blood, ≥ 5 cells	49	1.478 (0.734–2.974)	0.273		

*BMI* body mass index, *HBV* hepatitis B virus, *HCV* hepatitis C virus, *ICGR15* indocyanine green retention rate at 15 min, *AFP*α-fetoprotein, *DCP* des-γ-carboxy prothrombin, *CTC* circulating tumor cell

### Analysis of PD-L1 Expression on CTCs

**Fig. 3:**
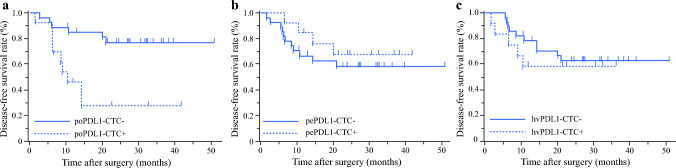
Survival after surgery by PD-L1-positive CTC count and site of collection. **a** Comparison of disease-free survival rates after hepatectomy of the poPD-L1-CTC^+^ and the poPD-L1-CTC^−^ groups. The disease-free survival rates were significantly lower in the poPD-L1-CTC^+^ group than in the poPD-L1-CTC^−^ group (*p* = 0.001). **b** Comparison of disease-free survival rates after hepatectomy between the pePD-L1-CTC^+^ and pePD-L1-CTC^−^ groups. There was no significant difference in disease-free survival rates between the pePD-L1-CTC^+^ and pePD-L1-CTC^−^ groups (*p* = 0.43). **c** Comparison of disease-free survival rates after hepatectomy between the hvPD-L1-CTC^+^ and hvPD-L1-CTC^−^ groups. There was no significant difference in disease-free survival rates between the hvPD-L1-CTC^+^ and hvPD-L1-CTC^−^ groups (*p* = 0.545)

To gain a deeper understanding of the involvement of poCTCs in PFS and OS, we evaluated PD-L1 expression in each CTC (from June 2020). Of the 40 patients evaluated for PD-L1 expression on poCTCs, 13 were positive for PD-L1. There were no significant differences in the backgrounds of the PD-L1-positive and -negative groups, except for a significantly lower body mass index in the positive group (Supplementary Table 8). Prognostic evaluation showed that the PD-L1-positive group had a significantly poorer DFS (*p* = 0.001; Fig. 3). Similarly, PD-L1 expression in peCTCs and hvCTCs was examined, with no significant effect on prognosis.

## Discussion

Liver resection is one of the most curative treatments for HCC; however, the prognosis after resection is poor in cases with microscopic PVI. HCC tends to invade the portal vein and is believed to metastasize through it to the liver and distant organs. Microscopic PVI is considered one of the strongest poor prognostic factors.^17^ In our study, portal vein CTCs were detected more frequently than hepatic vein CTCs, suggesting that CTCs may play an important role in microscopic PVI in HCC. Furthermore, in this study, CTCs were significantly associated with microscopic PVI, DFS, and OS, whereas microscopic PVI itself was not associated with DFS or OS. This indicates a strong confounding relationship between CTCs and microscopic PVI, which was resolved in multivariate analysis—suggesting that CTCs may serve as a superior prognostic indicator. In particular, poCTCs may more sensitively detect the presence of cancer cells in the portal circulation, potentially reflecting the true tumor burden more accurately than microscopic PVI, which identifies only tumor cells adherent to the portal vein wall.

Only two reports have investigated CTCs in the portal veins of HCC patients.^5,18^ Sun et al. assessed CTCs in the peripheral vein, peripheral artery, hepatic veins, infrahepatic inferior vena cava, and portal vein before tumor resection.^5^ The authors systematically mapped CTC distribution, characterized their epithelial and mesenchymal transition features, and evaluated their clinical significance across multiple vascular compartments in patients with localized HCC. CTCs and circulating tumor microemboli burden in the hepatic veins and peripheral circulation predicted postoperative lung metastasis and intrahepatic recurrence.^5^ In their study, the CTCs of each site were examined, but recurrence correlated only with CTCs in the hepatic vein and peripheral blood and not in portal vein blood. However, in the present study, portal vein blood CTCs were strongly associated with vascular invasion, recurrence, and prognosis, which was significantly different from this report. One factor contributing to this discrepancy is the use of GPC3, rather than EpCAM, for CTC detection. EpCAM-based detection may be insufficient to capture the prognostic significance of highly tumor-specific CTCs. EpCAM is expressed in approximately 35% of HCC cases.^19^ Furthermore, tumor cells that have undergone epithelial-to-mesenchymal transition (EMT) often show reduced EpCAM expression, potentially leading to the oversight of EMT-type CTCs. By contrast, GPC3 is highly expressed in HCC and functions as a promoter of EMT.^20^ Based on these observations, GPC3-positive CTCs detected in the portal vein may represent tumor-derived cells with metastatic potential that are directly identifiable within the vascular compartment. This may partially account for their strong association with DFS and OS. Given that GPC3 is also implicated in tumor invasiveness and stem cell-like characteristics, GPC3^+^ CTCs present in the portal vein may signify a population of highly malignant cells with a high potential for recurrence.

Zhao et al. reported that the portal vein CTC count before treatment correlated with vascular invasion and could be considered as one of the factors affecting HCC metastasis.^18^ They suggested that increased intrahepatic vascular resistance, chaotic blood flow in the tumor and liver parenchyma, and vascular invasion of tumor cells may provide a passage for CTCs into the portal vein.^18^ They demonstrated that portal vein CTCs were related to HCC metastasis. However, because not all patients underwent liver resection, they did not mention recurrence. In the present study, we further examined portal vein CTC in surgical cases and found that portal vein CTC was also related to HCC recurrence and survival.

In the present study, portal vein CTCs were associated with HCC recurrence and survival; however, it was challenging to collect portal vein blood. Portal vein blood can be collected during surgery; nonetheless, this procedure is not easy to perform preoperatively. Methods for preoperative portal vein blood collection include endoscopic ultrasonography-guided portal venous sampling or ultrasonography-guided transhepatic puncture.^21,22^ Although portal vein blood is more difficult to sample than peripheral blood, sampling using methods such as those described above has been reported.

We established a CTC detection method targeting GPC3, a cell surface marker specific to HCC, and previously reported its clinical utility.^9^ Consistent with earlier studies,^23–33^ we demonstrated that CTC positivity was an independent predictor of microscopic PVI. Tumor vascular invasion is also a strong risk factor for recurrence; however, it is practically impossible to evaluate the extent of invasion before surgery.^34^ In the present study, we observed a diagnostic discrepancy between macroscopic and microscopic PVI, with a sensitivity of only 0.152 for detecting microscopic PVI using macroscopic findings (Supplementary Table 5). This suggests that many cases of microvascular invasion remain undetected prior to surgery. In addition, many studies have reported that the presence of CTC is a negative prognostic factor for HCC.^24,28–31,35–39^ Similarly, the present study showed that patients in the CTC-positive group had poorer posthepatectomy DFS rates than did those in the CTC-negative group. Thus, CTCs may be useful biomarkers for HCC patient outcomes.

PD-L1-expressing CTCs in the portal vein had a significantly poorer prognosis than did nonexpressing cases. These results suggest the existence of a specific environment, such as immune escape, for CTC survival in portal blood. PD-L1 belongs to the B7 gene family and acts as a ligand for the programmed death receptor-1 (PD-1). It is found on the surfaces of cancer cells, dendritic cells, monocytes, and macrophages. Studies have shown that the binding of PD-1 to PD-L1 triggers a pathway that prevents T cells from activating.^18,40,41^ In HCC cells, PD-L1 was expressed at a rate of 23.9–81.1%.^42^ PD-L1 overexpression on the HCC cell surface is significantly associated with poorer OS and DFS.^43,44^ The molecular mechanisms by which PD-L1 promotes HCC progression include the following:

1. Inhibition of T-cell function: PD-L1 binds to PD-1 and transmits inhibitory signals that reduce T-cell receptor and CD28 activity. This leads to decreased T-cell activation and proliferation, ultimately allowing cancer cells to evade immune detection.^45^
2. Macrophage-mediated immune suppression: Macrophages play a crucial role in the tumor immune microenvironment. Research has shown that interleukin-4 activates macrophages, which in turn upregulate PD-L1 expression in tumor cells. This increase in PD-L1 expression promotes T-cell apoptosis.^46^
3. Inflammatory mechanism exploitation: Cancer cells can hijack inflammatory processes by inducing immune cells to secrete interferon-gamma. This cytokine then acts on hepatic nonparenchymal cells, stimulating them to express PD-L1 on their surfaces.^47,48^

Based on these findings, Zhao et al. suggested that PD-L1 functions as a protumorigenic factor and may serve as an important biomarker for HCC with microvascular invasion.^49^ Our results suggested that the presence of PD-L1-expressing CTCs in the portal vein was associated with poor outcomes. This adverse prognosis likely results from the combination of enhanced immune evasion and increased metastatic potential. Moving forward, it would be valuable to analyze the correlation between PD-L1–expressing CTCs and other clinical parameters (e.g., tumor size, metastasis, and treatment response) to establish a more precise risk evaluation and develop individualized treatment strategies. Furthermore, improving the detection methods for PD-L1-expressing CTCs and monitoring their dynamics during therapy could offer additional insights into therapeutic decision-making.

This study had some limitations. First, liver resection cases with portal vein blood sampling were limited to anatomical resections, excluding partial resections. Therefore, there is a bias towards HCC that requires anatomical resection in these results. Second, PD-L1 expression in primary tumors has not yet been confirmed. By comparing the expression of PD-L1 in primary tumors with that in CTCs, we may be able to better understand PD-L1 expression. If the expression of PD-L1 in the primary tumor and CTCs is correlated, the characteristics of the primary tumor and CTCs are consistent. Therefore, it may be of low significance to investigate PD-L1 expression in CTCs. However, if the expression is not correlated, it may be important to investigate PD-L1 expression in CTCs.

## Conclusion

We showed that the presence of CTCs in the portal blood is associated with microscopic PVI, recurrence, and death in patients with HCC. Additionally, PD-L1 expression in portal CTCs is significantly associated with poor patient prognosis. These results suggested that cancer cells invading the portal vein may undergo immune escape.

## Supplementary Information

Below is the link to the electronic supplementary material.

Supplementary file1 (DOCX 18 KB)

Supplementary file2 (DOCX 18 KB)

Supplementary file3 (DOCX 18 KB)

Supplementary file4 (DOCX 15 KB)

Supplementary file5 (DOCX 15 KB)

Supplementary file6 (DOCX 16 KB)

Supplementary file7 (DOCX 16 KB)

Supplementary file8 (DOCX 18 KB)

Supplementary Fig. 1 Flow chart of the participant selection (TIF 104 KB)

Supplementary Fig. 2 Dot and box plots of GPC3-positive CTC count. Distribution of GPC3-positive CTCs in patients with HCC (*n* = 146) who underwent anatomical hepatectomy. The number of CTCs was three (0–77) in the peripheral blood, four (0–43) in the portal vein, and three (0–51) in the hepatic vein. The number of CTCs was significantly higher in the portal vein than in the hepatic vein (*p* < 0.05) (TIF 80 KB)

Supplementary Fig. 3. ROC curve of peCTC count for prediction of microscopic portal vein invasion in HCC patients (AUC = 0.727; cutoff = 5). **a** ROC curve of poCTC count for prediction of microscopic portal vein invasion in HCC patients (AUC = 0.718; cutoff = 5). **b** ROC curve of hvCTC count for prediction of microscopic portal vein invasion in pHCC patients (AUC = 0.563; cutoff = 6) (TIF 135 KB)
