# Supplementary material for: Clinical Significance of Circulating Tumor Cells in the Portal Vein of Patients with Hepatocellular Carcinoma Undergoing Anatomical Liver Resection
Source: Ann Surg Oncol. 2025 Sep 9;32(13):9561–72. doi: 10.1245/s10434-025-18295-5 (PMC12589225; doi:10.1245/s10434-025-18295-5)
Supplement: Supplementary file 1 — Supplementary file1 (DOCX 18 KB) [file 10434_2025_18295_MOESM1_ESM.docx]

Supplementary Table 1. Patients’ characteristics (n=146).

| Variables | Number of patients (%) |
| --- | --- |
| Age (years)^＊^ | 72.5 (48-89) |
| Male | 115 (78.7) |
| BMI (kg/m^2^)^＊^ | 23.2 (16.7-33.1) |
| HBV | 16 (10.9) |
| HCV | 60 (41.0) |
| ICGR15 (%)^＊^ | 12.0 (2.5-41.1) |
| Child-Pugh grade B | 8 (5.4) |
| AFP (ng/mL)^＊^ | 6.5 (0.7-290700) |
| DCP (mAU/mL)^＊^ | 131 (3.4-124040) |
| Preoperative TACE | 11 (7.5) |
| Preoperative RFA | 1 (0.6) |
| Number of tumors^＊^ | 1 (1-20) |
| Tumor size (mm)^＊^ | 35 (10-200) |
| Macroscopic portal vein invasion: n (%) | 6 (4.1) |
| Macroscopic hepatic vein invasion: n (%) | 7 (4.7) |
| Microscopic portal vein invasion: n (%) | 33 (22.6) |
| Microscopic hepatic vein invasion: n (%) | 15 (10.2) |
| Tumor differentiated  well  moderate  poorly | 20 (13.6)  107 (73.2)  19 (13.2) |
| Surgical procedure  Segmentectomy  Sectionectomy  Bi-segmentectomy  Lobectomy | 92 (63.0)  32 (21.9)  2 (1.3)  20 (13.6) |
| Number of CTCs in peripheral blood (cells)^＊^ | 3 (0-77) |
| Number of CTCs in portal vein blood (cells)^＊^ | 4 (0-43) |
| Number of CTCs in hepatic vein blood (cells)^＊^ | 3 (0-51) |

^＊^Median (range)

BMI : Body Mass Index; HBV: hepatitis B virus; HCV: hepatitis C virus; ICGR15: indocyanine green retention rate at 15 min; AFP: α-fetoprotein; DCP: des-γ-carboxy prothrombin; CTC: circulating tumor cell; TACE: transarterial chemoembolization; RFA: radiofrequency ablation
