# Supplementary material for: Clinical Significance of Circulating Tumor Cells in the Portal Vein of Patients with Hepatocellular Carcinoma Undergoing Anatomical Liver Resection
Source: Ann Surg Oncol. 2025 Sep 9;32(13):9561–72. doi: 10.1245/s10434-025-18295-5 (PMC12589225; doi:10.1245/s10434-025-18295-5)
Supplement: Supplementary file 2 — Supplementary file2 (DOCX 18 KB) [file 10434_2025_18295_MOESM2_ESM.docx]

Supplementary Table 2. Clinicopathologic features between the peCTC ≧ 5 and < 5 groups

|  | peCTC ≧ 5  (n=54) | peCTC < 5  (n=92) | p-value |
| --- | --- | --- | --- |
| Age (year)^＊^ | 70.5 (48-85) | 74 (48-89) | 0.008 |
| Male: n (%)^＊^ | 44 (81%) | 71 (77%) | 0.538 |
| BMI (kg/m^2^)^＊^ | 23.8 (16.8-33.1) | 23.0 (16.7-32.2) | 0.324 |
| HBV: n (%) | 7 (13%) | 9 (10%) | 0.552 |
| HCV: n (%) | 21 (39%) | 39 (42%) | 0.678 |
| ICGR15 (%)^＊^ | 12.6 (2.6-41.1) | 11.6 (2.5-34.2) | 0.521 |
| Child-Pugh grade B: n (%) | 4 (7%) | 4 (4%) | 0.432 |
| AFP (ng/mL)^＊^ | 10.4 (1.6-23789) | 5.2 (0.7-290700) | 0.104 |
| DCP (mAU/mL)^＊^ | 317 (3.4-124040) | 115 (10-54832) | 0.206 |
| Tumor number^＊^ | 1 (1-4) | 1 (1-20) | 0.026 |
| Tumor size (mm)^＊^ | 39 (10-200) | 30 (10-145) | 0.017 |
| Macroscopic portal vein invasion: n (%) | 5 (9%) | 1 (1%) | 0.016 |
| Macroscopic hepatic vein invasion: n (%) | 4 (7%) | 3 (3%) | 0.257 |
| Microscopic portal vein invasion: n (%) | 23 (43%) | 10 (11%) | <0.001 |
| Microscopic hepatic vein invasion: n (%) | 10 (19%) | 5 (5%) | 0.011 |
| Major hepatectomy | 11 (20%) | 11 (12%) | 0.170 |
| Surgical margin (mm)^＊^ | 2.5 (0-30) | 3 (0-40) | 0.483 |
| Estimated blood loss (g)^＊^ | 524 (35-3385) | 491 (20-3242) | 0.257 |

＊Median (range)

peCTC : peripheral blood circulating tumor cell; BMI : Body Mass Index; HBV: hepatitis B virus; HCV: hepatitis C virus; ICGR15: indocyanine green retention rate at 15 min; AFP: α-fetoprotein; DCP: des-γ-carboxy prothrombin
