# Supplementary material for: Clinical Significance of Circulating Tumor Cells in the Portal Vein of Patients with Hepatocellular Carcinoma Undergoing Anatomical Liver Resection
Source: Ann Surg Oncol. 2025 Sep 9;32(13):9561–72. doi: 10.1245/s10434-025-18295-5 (PMC12589225; doi:10.1245/s10434-025-18295-5)
Supplement: Supplementary file 3 — Supplementary file3 (DOCX 18 KB) [file 10434_2025_18295_MOESM3_ESM.docx]

Supplementary Table 3. Clinicopathologic features between the hvCTC ≧ 5 and < 5 groups

|  | hvCTC ≧ 5  (n=49) | hvCTC < 5  (n=97) | p-value |
| --- | --- | --- | --- |
| Age (year)^＊^ | 70 (53-87) | 74 (48-89) | 0.064 |
| Male: n (%)^＊^ | 41 (84%) | 74 (76%) | 0.302 |
| BMI (kg/m^2^)^＊^ | 22.9 (16.7-29.1) | 23.4 (16.8-33.1) | 0.502 |
| HBV: n (%) | 7 (14%) | 9 (9%) | 0.360 |
| HCV: n (%) | 15 (30%) | 45 (46%) | 0.067 |
| ICGR15 (%)^＊^ | 10.6 (2.6-41.1) | 12.6 (2.5-37.7) | 0.146 |
| Child-Pugh grade B: n (%) | 3 (6%) | 5 (5%) | 0.808 |
| AFP (ng/mL)^＊^ | 6.4 (1.6-23789) | 6.9 (0.7-290700) | 0.657 |
| DCP (mAU/mL)^＊^ | 147 (3.4-124040) | 130 (10-56162) | 0.991 |
| Tumor number^＊^ | 1 (1-20) | 1 (1-6) | 0.129 |
| Tumor size (mm)^＊^ | 35 (12-200) | 30 (10-145) | 0.341 |
| Macroscopic portal vein invasion:  n (%) | 4 (8%) | 2 (2%) | 0.079 |
| Macroscopic hepatic vein invasion: n (%) | 3 (6%) | 4 (4%) | 0.593 |
| Microscopic portal vein invasion:  n (%) | 14 (29%) | 19 (20%) | 0.220 |
| Microscopic hepatic vein invasion: n (%) | 5 (10%) | 10 (10%) | 0.984 |
| Major hepatectomy: n (%) | 10 (20%) | 12 (12%) | 0.199 |
| Surgical margin (mm)^＊^ | 3 (0-30) | 3 (0-40) | 0.672 |
| Estimated blood loss (g)^＊^ | 511 (54-3385) | 522 (20-3242) | 0.752 |

＊Median (range)

hvCTC : hepatic vein circulating tumor cell; BMI : Body Mass Index; HBV: hepatitis B virus; HCV: hepatitis C virus; ICGR15: indocyanine green retention rate at 15 min; AFP: α-fetoprotein; DCP: des-γ-carboxy prothrombin
