# Supplementary material for: Clinical Significance of Circulating Tumor Cells in the Portal Vein of Patients with Hepatocellular Carcinoma Undergoing Anatomical Liver Resection
Source: Ann Surg Oncol. 2025 Sep 9;32(13):9561–72. doi: 10.1245/s10434-025-18295-5 (PMC12589225; doi:10.1245/s10434-025-18295-5)
Supplement: Supplementary file 4 — Supplementary file4 (DOCX 15 KB) [file 10434_2025_18295_MOESM4_ESM.docx]

Supplementary Table 4. Comparison of CTC counts according to the presence of macroscopic PVI

|  | Macroscopic PVI  Positive (n=6) | Macroscopic PVI  Negative (n=140) | p-value |
| --- | --- | --- | --- |
| CTC count in peripheral blood | 10.5 (1-18) | 3 (0-77) | 0.031 |
| CTC count in portal vein blood | 8.5 (1-43) | 4 (0-33) | 0.103 |
| CTC count in hepatic vein blood | 5 (2-14) | 3 (0-51) | 0.113 |

＊Median (range)

CTC: circulating tumor cell; PVI: portal vein invasion
