# Supplementary material for: Clinical Significance of Circulating Tumor Cells in the Portal Vein of Patients with Hepatocellular Carcinoma Undergoing Anatomical Liver Resection
Source: Ann Surg Oncol. 2025 Sep 9;32(13):9561–72. doi: 10.1245/s10434-025-18295-5 (PMC12589225; doi:10.1245/s10434-025-18295-5)
Supplement: Supplementary file 5 — Supplementary file5 (DOCX 15 KB) [file 10434_2025_18295_MOESM5_ESM.docx]

Supplementary Table 5. Relationship between microscopic and macroscopic PVI in HCC

|  | Microscopic PVI  Positive (n=33) | Microscopic PVI  Negative (n=113) |
| --- | --- | --- |
| Macroscopic PVI  Positive (n=6) | 5 | 1 |
| Macroscopic PVI  Negative (n=140) | 28 | 112 |

PVI: portal vein invasion; HCC: hepatocellular carcinoma
