# Supplementary material for: Clinical Significance of Circulating Tumor Cells in the Portal Vein of Patients with Hepatocellular Carcinoma Undergoing Anatomical Liver Resection
Source: Ann Surg Oncol. 2025 Sep 9;32(13):9561–72. doi: 10.1245/s10434-025-18295-5 (PMC12589225; doi:10.1245/s10434-025-18295-5)
Supplement: Supplementary file 6 — Supplementary file6 (DOCX 16 KB) [file 10434_2025_18295_MOESM6_ESM.docx]

|  | Microscopic PVI positive  (n=33) | Microscopic PVI negative  (n=113) | Macroscopic PVI positive  (n=6) | Macroscopic PVI negative  (n=140) |
| --- | --- | --- | --- | --- |
| peCTC positivity, n (%) | 23 (70%) | 31 (27%) | 5 (83%) | 49 (35%) |
| poCTC positivity, n (%) | 25 (76%) | 42 (37%) | 5 (83%) | 62 (44%) |
| hvCTC positivity, n (%) | 14 (42%) | 35 (31%) | 4 (67%) | 45 (32%) |

Supplementary Table 6. Comparison of CTC positivity according to the presence of microscopic and macroscopic PVI

CTC: circulating tumor cell; PVI: portal vein invasion; peCTC: peripheral vein CTC; poCTC: portal vein CTC; hvCTC: hepatic vein CTC
