# Supplementary material for: Clinical Significance of Circulating Tumor Cells in the Portal Vein of Patients with Hepatocellular Carcinoma Undergoing Anatomical Liver Resection
Source: Ann Surg Oncol. 2025 Sep 9;32(13):9561–72. doi: 10.1245/s10434-025-18295-5 (PMC12589225; doi:10.1245/s10434-025-18295-5)
Supplement: Supplementary file 7 — Supplementary file7 (DOCX 16 KB) [file 10434_2025_18295_MOESM7_ESM.docx]

Supplementary Table 7. Comparison of CTC number according to the presence of microscopic PVI and HVI

|  | Both PVI and HVI  (n=10) | Only mPVI  (n=23) | Only mHVI  (n=5) | None  (n=108) | p-value |
| --- | --- | --- | --- | --- | --- |
| CTCs in peripheral blood | 5.5 (4.5-11.25) | 5 (3-9) | 2 (0.5-6) | 2 (1-5) | 0.001 |
| CTCs in  portal vein blood | 9 (2-16.75) | 7 (5-16) | 1 (0-11) | 3 (1-6) | 0.001 |
| CTCs in  hepatic vein blood | 2 (0-6.5) | 3 (2-7) | 1 (0-4) | 3 (1-5.75) | 0.341 |

＊Median (range)

CTC: circulating tumor cell; PVI: portal vein invasion; HVI: hepatic vein invasion; mPVI: microscopic PVI; mHVI: microscopic HVI
