# Supplementary material for: Clinical Significance of Circulating Tumor Cells in the Portal Vein of Patients with Hepatocellular Carcinoma Undergoing Anatomical Liver Resection
Source: Ann Surg Oncol. 2025 Sep 9;32(13):9561–72. doi: 10.1245/s10434-025-18295-5 (PMC12589225; doi:10.1245/s10434-025-18295-5)
Supplement: Supplementary file 8 — Supplementary file8 (DOCX 18 KB) [file 10434_2025_18295_MOESM8_ESM.docx]

|  | PDL1^+^ poCTC (n=13) | PDL1^-^ poCTC  (n=27) | p-value |
| --- | --- | --- | --- |
| Age (year) | 80 (67-89) | 75 (53-84) | 0.151 |
| Male: n (%) | 11 (85%) | 19 (70%) | 0.329 |
| BMI (kg/m^2^) | 21.8 (16.8-26.8) | 24.6 (19.1-29.0) | 0.029 |
| HBV: n (%) | 1 (8%) | 5 (19%) | 0.369 |
| HCV: n (%) | 6 (46%) | 7 (26%) | 0.200 |
| ICGR15 (%) | 11.5 (3.1-19.7) | 11.1 (4.5-24.9) | 0.772 |
| Child-Pugh grade B: n (%) | 1 (8%) | 1 (4%) | 0.587 |
| AFP (ng/mL) | 7.8 (0.9-295) | 4.5 (1.5-462) | 0.435 |
| DCP (mAU/mL) | 270 (29-33365) | 81 (11-53668) | 0.133 |
| Tumor number | 1 (1-3) | 1 (1-6) | 0.945 |
| Tumor size (mm) | 43 (20-80) | 35 (10-120) | 0.582 |
| Microscopic portal vein invasion:  n (%) | 3 (23%) | 3 (11%) | 0.320 |
| Microscopic hepatic vein invasion: n (%) | 1 (8%) | 3 (11%) | 0.735 |
| Number of CTCs in peripheral blood (cells) | 1 (0-9) | 3 (0-76) | 0.301 |
| Number of CTCs in portal vein blood (cells) | 5 (1-20) | 1 (0-16) | 0.012 |
| Number of CTCs in hepatic vein blood (cells) | 2 (0-7) | 2 (0-22) | 0.836 |

Supplementary Table 8. Clinicopathologic features between PDL1^+^ poCTC and PDL1^-^ poCTC groups

PDL1 : programmed cell death ligand 1; poCTC : portal vein circulating tumor cell; BMI : Body Mass Index; HBV: hepatitis B virus; HCV: hepatitis C virus; ICGR15: indocyanine green retention rate at 15 min; AFP: α-fetoprotein; DCP: des-γ-carboxy prothrombin; CTC: circulating tumor cell
