# Supplementary figures and images for: Clinical Significance of Circulating Tumor Cells in the Portal Vein of Patients with Hepatocellular Carcinoma Undergoing Anatomical Liver Resection
Source: Ann Surg Oncol. 2025 Sep 9;32(13):9561–72. doi: 10.1245/s10434-025-18295-5 (PMC12589225; doi:10.1245/s10434-025-18295-5)

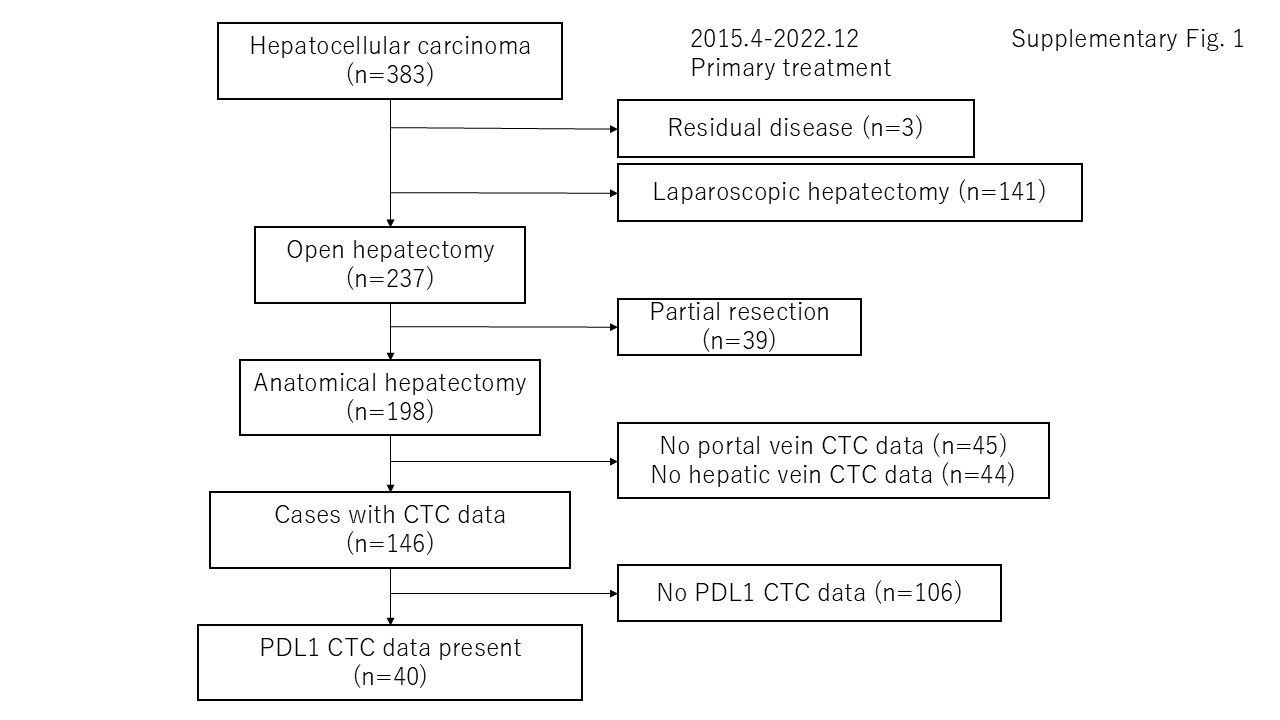

Supplement: Supplementary file 9 — Supplementary Fig. 1 Flow chart of the participant selection (TIF 104 KB) [file 10434_2025_18295_MOESM9_ESM.tif]

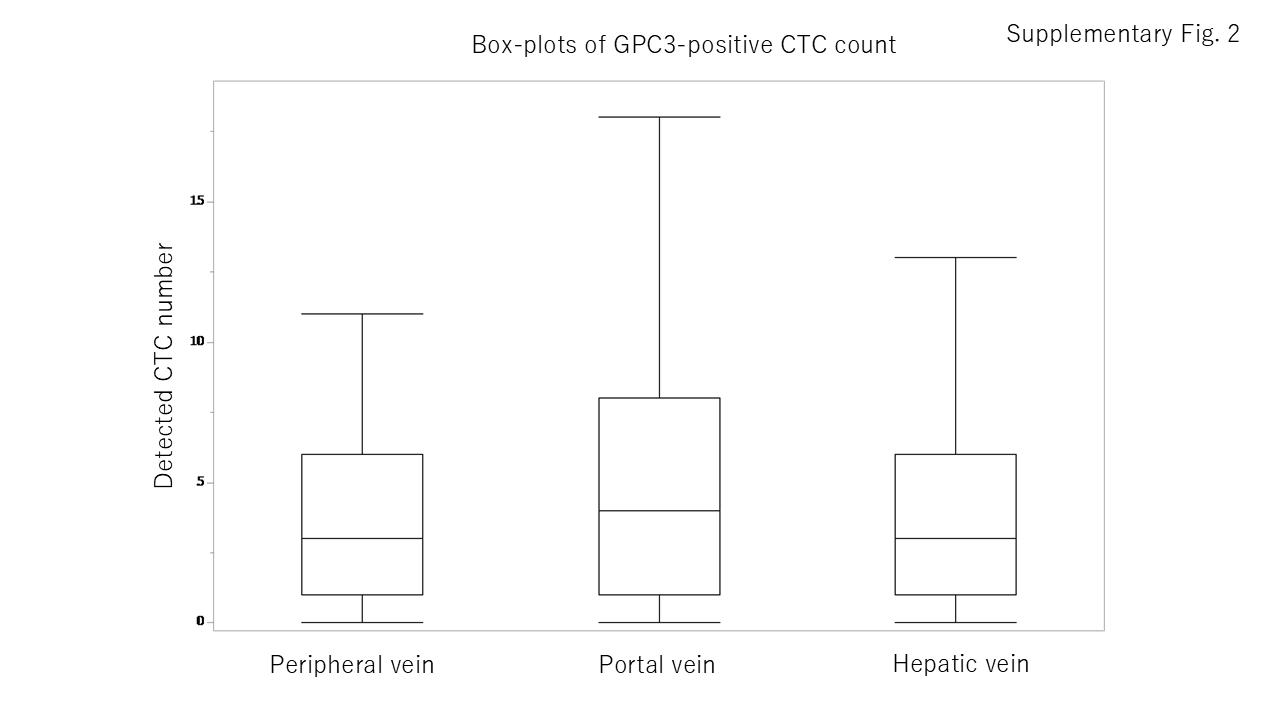

Supplement: Supplementary file 10 — Supplementary Fig. 2 Dot and box plots of GPC3-positive CTC count. Distribution of GPC3-positive CTCs in patients with HCC (n = 146) who underwent anatomical hepatectomy. The number of CTCs was three (0–77) in the peripheral blood, four (0–43) in the portal vein, and three (0–51) in the hepatic vein. The number of CTCs was significantly higher in the portal vein than in the hepatic vein (p < 0.05) (TIF 80 KB) [file 10434_2025_18295_MOESM10_ESM.tif]

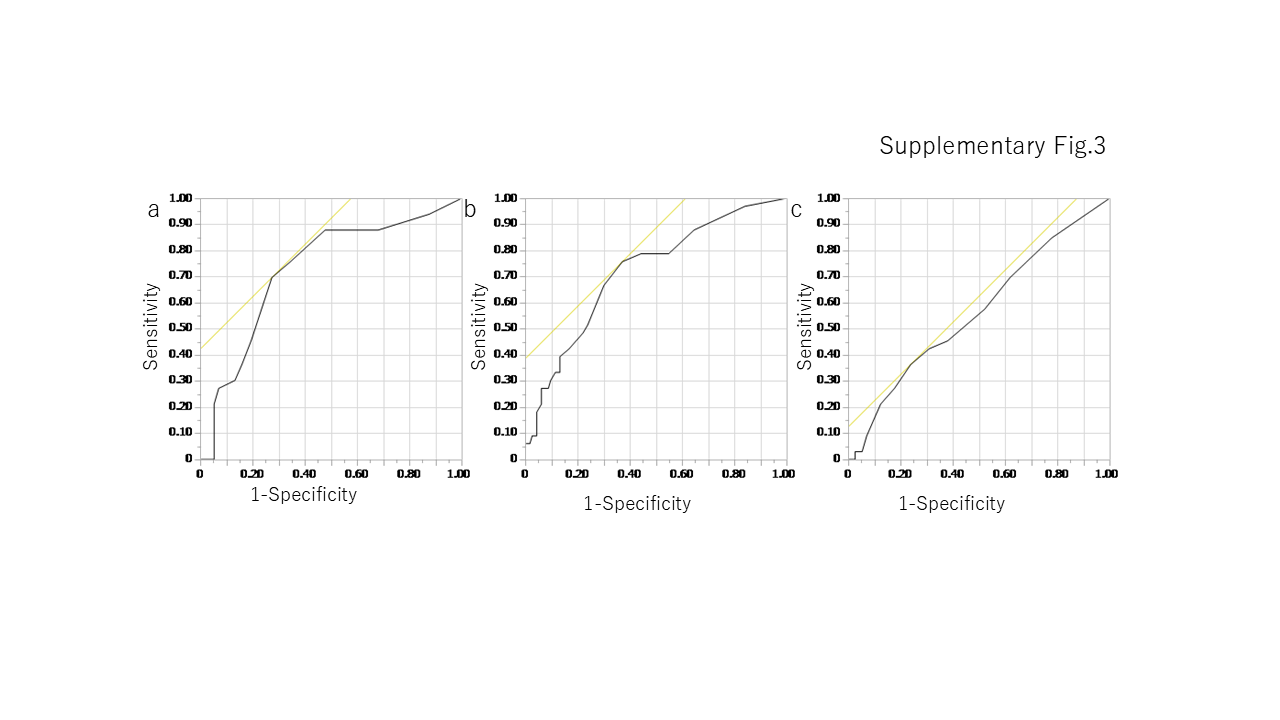

Supplement: Supplementary file 11 — Supplementary Fig. 3. ROC curve of peCTC count for prediction of microscopic portal vein invasion in HCC patients (AUC = 0.727; cutoff = 5). a ROC curve of poCTC count for prediction of microscopic portal vein invasion in HCC patients (AUC = 0.718; cutoff = 5). b ROC curve of hvCTC count for prediction of microscopic portal vein invasion in pHCC patients (AUC = 0.563; cutoff = 6) (TIF 135 KB) [file 10434_2025_18295_MOESM11_ESM.tif]
